# Establishment of human trabecular meshwork cell cultures using nontransplantable corneoscleral rims

**DOI:** 10.3906/biy-1810-69

**Published:** 2019-04-05

**Authors:** Kosala D. WADUTHANTHRI, Carlo MONTEMAGNO, Sibel ÇETİNEL

**Affiliations:** 1 Ingenuity Lab, Department of Chemical and Materials Engineering, University of Alberta , Edmonton, AB , Canada; 2 Southern Illinois University , Carbondale, IL , USA

**Keywords:** Trabecular meshwork, primary cell culture, dexamethasone, phagocytosis, Optisol-GS

## Abstract

Human trabecular meshwork (hTM) cell isolation in academic settings utilizes the motile nature of these cells, allowing them to migrate away from the explant and proliferate on distal regions of the culture substrate. Corneoscleral rims used for transplantation are a potential source of explants for the establishment of hTM cell cultures. However, cell isolation and the initiation of primary cell cultures from ocular tissues stored in Optisol-GS medium for an extended period of time (>6 days) has proven difficult, since Optisol-GS remarkably reduces cell viability and cellularity. Therefore, explants obtained from ocular tissues stored in Optisol-GS do not often provide adequate cell yield to initiate primary cell cultures if conventional culture techniques are used. Therefore, the majority of the research on primary hTM cell isolation has been accomplished using donor tissue obtained within 72 h postmortem. The goal of this study was to develop an hTM cell isolation procedure from nontransplantable ocular materials, utilizing the anchorage dependency of TM cells. This procedure yielded functionally viable cells, eficiently dissociated from the trabecular meshwork. Isolated cells demonstrated typical hTM cell characteristics including monolayer formation, contact inhibition, phagocytosis, and responses to glucocorticoid exposure. To the best of our knowledge, this is the first time an expired explant has been utilized in the successful isolation of hTM cells. Our results clearly demonstrate the advantage of increasing the anchor points of hTM cells for enhanced cell migration out from the explants, which have limited cell proliferative capacity.

## 1. Introduction

The human trabecular meshwork (hTM), located at the iridocorneal angle, is an intricate 3D structure composed of a collagenous and elastin-like extracellular matrix (ECM) in which trabecular meshwork (TM) cells are embedded (Stamer and Clark, 2017) . These cells specialize in the production, maintenance, and modification of the ECM, keeping aqueous humor drainage through the conventional outflow pathway at an optimum level and thereby keeping intraocular pressure (IOP) at physiological level (Tamm, 2009) . Aqueous humor, secreted by the ciliary epithelium, propels through the TM into Schlemm’s canal (SC), where it travels through collector channels into the episcleral veins (Dautriche et al., 2014) . In a healthy eye, aqueous humor production is relatively constant and IOP remains within a narrow range thanks to the modulation of outflow rate though the TM (Dautriche et al., 2014) .

The hTM can be anatomically divided into three differentiated layers depending on architectural complexity. These are, from the inner to outermost layer, the uveal, corneoscleral, and juxtacanalicular tissue (JCT) regions (Tamm, 2009) . The uveal meshwork consists of trabecular beams composed of a core of collagen and elastin covered by a basal lamina rich in laminin and collagen type IV. The trabecular beams are arranged in several layers, creating intratrabecular spaces in a fenestrated structure, through which aqueous humor flows (Dautriche et al., 2014) . The corneoscleral meshwork contains more trabecular layers, thicker than those seen in the uveal meshwork. The pore size of the tissue becomes progressively smaller as it extends closer to the SC. The third layer, the JCT, also known as the cribriform region, is located directly adjacent to the inner wall of the SC (Tamm, 2009) . The JCT does not form trabecular lamellae or beams, but is composed of a loosely arranged fibrillar extracellular matrix. The JCT cells are in contact with each other as well as with the endothelial cell lining of the SC and other TM beam cells via long cytoplasmic processes (McEwen, 1957) .

TM cells residing in the aqueous humor outflow facility exhibit two different morphologies even though they have a common embryonic origin, the neural crest (Tripathi and Tripathi, 1982) . Specifically, cells derived from the uveal and corneoscleral layers are round to oval in shape and have an endothelial-like morphology (Stamer and Clark, 2017) . These aggressively phagocytic cells ingest cellular debris and pigment granules derived from epithelial turnover events. The inner TM rapidly clears this cellular debris before it reaches the deeper TM regions and creates the risk of accumulation and increased outflow resistance. Additionally, endothelial-like TM cells help to sustain healthy aqueous humor outflow by producing antithrombotic substances. Therefore, uveal and corneoscleral regions can be described as biological iflters. Cells derived from the outer JCT exhibit spindle shape morphology with fibroblastic and smooth-musclelike characteristics (Stamer and Clark, 2017) . These cells secrete large quantities of ECM proteins and remodel the ECM by degrading its components in order to maintain the TM’s complex structural organization at an optimal level (Keller et al., 2009) . TM cells in the JCT and corneoscleral region are also contractile with the production of α-smooth muscle actin and myocilin. In general, the JCT together with the corneoscleral region is responsible for resistance generation. It is believed that pathophysiological conditions alter the ability of TM cells to modulate the ECM structure, resulting in increased resistance to aqueous humor (Vranka et al., 2015) .

TM cell culture systems would provide a valuable way to study TM cell physiology as well as obtain cells to establish a model system for the study of glaucoma (Rybkin et al., 2017) . Furthermore, it would be a valuable source for the reconstruction of TM in tissue engineering applications. Rohen et al. (1975) first reported the successful establishment of primary TM culture using dissected TM explants obtained from long-tailed monkeys. Following that, several research groups successfully isolated hTM cells from explants dissected from postmortem eyes and reported characteristic morphological and functional features including an elongated appearance with long cellular processes, the presence of apical villous, fibronectin secretion, phagocytic activity, etc. (Polansky et al., 1979; Alvarado et al., 1982; Tripathi and Tripathi, 1982; Keller et al., 2018) . Over the past few decades, a significant improvement has been made in culturing TM cells from various sources with the aid of advanced cell culture techniques. However, establishing a primary TM cell culture from tissues obtained from nonfresh samples is still challenging in practice due to the loss of viability of TM cells during tissue storage (Keller et al., 2018) . In addition, TM explants obtained from older donors do not often provide the cell yield required for the establishment of a primary cell culture, due to limited cell proliferative capacity.

Corneoscleral rims used for transplantation are a potential source of TM explants for the establishment of primary TM cell cultures (Rhee et al., 2003) . However, the viability and the number of cells isolated from these tissues using conventional techniques are limited, as these tissues are stored in an Optisol medium for several days. Therefore, to establish TM cell cultures, researchers often use tissues obtained within 24 h postmortem or tissues stored in Optisol for less than 7 days (Keller et al., 2018) . In this study, we are reporting a successful approach for isolating TM cells from nonfresh corneoscleral tissue sources, utilizing the anchorage dependency of TM cells.

## 2. Materials and methods

Human trabecular meshwork cells were isolated from the corneoscleral rim tissues of two donors (age 58 and 66) unsuitable for transplantation (obtained from the Comprehensive Tissue Centre, Alberta Health Services, Edmonton, Alberta, Canada) under the approval of the Health Research Ethics Boards at the University of Alberta (Study ID: Pro00059371). The corneoscleral rims were stored in Optisol-GS (Bausch & Lomb Incorporated, Rochester, NY, USA) for 14 days before the expiry date for transplantation and TM isolation was done 1–2 days from the expiry date. All experiments involving human tissue/cells were performed in compliance with the tenets of the Declaration of Helsinki. The hTM cells were grown in Trabecular Meshwork Cell Media (TMCM) purchased from ScienCell (Carlsbad, CA, USA). TMCM consisted of a basal medium, fetal bovine serum, growth supplement, and penicillin/streptomycin. Unless specifically stated otherwise, the cells were maintained in a humidified atmosphere of 5% CO2 and 95% air, with media changes every other day.

### 2.1. hTM cell isolation and culture establishment

**Figure 1:**
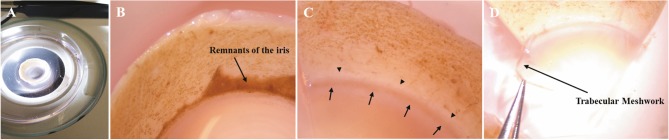
Series of images showing the removal of hTM from a donor tissue. (A) Corneoscleral rim placed in a petri dish. (B) Micrograph of a section of the anterior segment showing remnants of the iris attached to the sclera. (C) Micrograph of a tissue wedge of the anterior segment after removing iris remnants. Arrows: TM showing variable pigmentation in dark color. Arrowheads: Scleral spur. (D) Micrograph showing the removal of TM tissue as a strip using jeweler’s forceps.

Trabecular meshwork explants were obtained under direct observation with a dissection microscope, taking sterile precautions. The anterior segment was placed in a sterile petri plate and the remnants of the iris were gently pulled away from the cornea (Figures 1A–1C) using a pair of fine-tooth forceps, taking care to avoid damage to the angle area. Each anterior segment was then dissected into quadrants using a sharp razor blade. Each segment was washed in culture medium and the scleral spur was identified by its whitish luster. The TM was gently lifted away from the SC using sharp jeweler-type forceps, moving it from one end to the other end of the quadrant (Figure 1D). Isolated TM fragments were washed several times with the culture medium in order to remove any extraneous tissue fragments.

**Figure 2:**
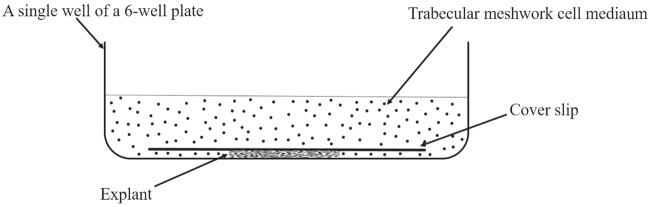
Schematic diagram of the well of a 6-well polystyrene plate, where TM explant is sandwiched between the gelatin-coated plastic bottom and the coverslip.

Some of the explants were digested with 100 µg/mL collagenase at 37 °C for 30 min to disrupt the connections between ECM and TM cells, which facilitates the movement of cells onto the culture surface (Stamer et al., 1995, 2000) . Samples were then centrifuged at 1500 × g for 5 min, the supernatants were removed by aspiration, and the cell pellets were resuspended in TMCM. Explants were then placed either in wells of gelatin-coated 6-well plates or sandwiched between the gelatin-coated bottom of a 6-well plate and gelatin-coated coverslips (Figure 2).Some of the explants were processed in a similar way, but without collagenase digestion.

Each well of 6-well plates containing the explant was supplemented with 2 mL of TMCM and the plates were maintained in a humidified atmosphere of 5% CO 2 and 95% air, with no media changes for 2 weeks until the tissue adhered to the substrate, and then fresh media were provided every 2 days. Once the established primary culture on the bottom of the 6-well plate was nearly conuflent, the coverslip was gently lifted and placed on the bottom of a gelatin-coated T-25 flask to allow the primary culture to expand. The coverslip was then covered with 4 mL of TMCM without disturbing the TM cell layer. Thereafter, the culture medium was renewed every other day until the flask became conuflent.

### 2.2. Immunohistochemistry analysis of hTM cells

Immunohistochemistry analyses were performed to detect the expression of selected cytoskeletal and extracellular matrix proteins. Unless specified otherwise, all primary and secondary antibodies were purchased from Abcam (Cambridge, MA, USA). Briefly, the hTM cells (passage 0 or 1) were plated onto gelatin-coated coverslips. Upon reaching conuflence (>90%), the coverslips were fixed with 4% paraformaldehyde for 20 min, washed with PBS three times, blocked with blocking medium (5% normal goat serum and 0.2% Triton X-100 in PBS) for 2 h, and incubated with the primary antibody (1:200 dilution in the blocking medium) overnight at 4 °C. The primary antibodies used were rabbit anticollagen IV, rabbit antifibronectin, and rabbit antimyocilin. The cells were washed three times in PBS to remove unbound primary antibodies and incubated with the secondary antibody, goat antirabbit IgG H&L (1:500 dilution in blocking medium), for 2 h. The cells were then washed three times with PBS and incubated with a 1:40 dilution (in PBS) of Alexa Fluor 488 Phalloidin (Thermo Fisher Scientific) for 20 min in order to stain F-actins. Preparations were mounted with DAPI-mounting solution (using a fluoroshield mounting medium with DAPI; Abcam, Cambridge, MA, USA) and viewed under an Olympus IX81 fluorescent microscope.

### 2.3. Evaluation of phagocytic activity

hTM cells (passage 1) were cultured on gelatin coated cover-slides in 6-well plates. Meanwhile, Staphylococcus aureus  bioparticles were opsonized according to the manufacturer’s instructions (Molecular Probes, Eugene, OR, USA) at 37 °C for 1 h, followed by three PBS washes. When the cultures reached semiconfluency, cells were challenged with opsonized Staphylococcus aureus  bioparticle-Alexa Fluor 488 conjugates (Thermo Scientific) at 50 bioparticles per cell concentration. At 0 and 3 h, cells being incubated at 37 °C were treated with 250 µg/mL Trypan blue (pH 7.2) for 2 min, followed by three PBS washes to remove free bioparticles. Preparations were then fixed in 4% paraformaldehyde solution for 20 min, mounted with DAPI-mounting solution, and imaged using an Olympus IX81 fluorescent microscope.

### 2.4. Cell proliferation rate and doubling time

hTM cells (passage 1) were cultured on gelatin-coated cover-slides in 6-well plates. The initial number of cells (N0) was determined 24 h after cell seeding. Three biological replicates, each with an average of three technical replicates, were used to obtain N0. Cells were then allowed to grow in TMCM for 3 days (time = t) and the number of cells (N) was counted similarly. Assuming that the growth rate is proportional to the number of cells, proliferation constant kP was determined using the following equation:

N=N0ekpt

The doubling time for the isolated cells was calculated using the following equation:

tD=ln2kp

where t_D_ is the doubling time of hTM cells, calculated after t time.

### 2.5. Dexamethasone (Dex) treatment

hTM cells cultured as monolayers in 6-well polystyrene plates were treated with 100 nM cell culture grade Dex (Sigma Aldrich) in fresh TMCM when monolayers were nearly 80% conuflent. Equivolume treatments of ethanol were used as the vehicle control. Cells were treated with either Dex or ethanol for 5 days before extracting RNA and the medium containing Dex or vehicle was changed daily. hTree replicates consisting of individual sets of treated and control cells were prepared for each treatment (n = 3).

### 2.6. Determination of MYOC gene expression by quantitative RT-PCR

Total RNA was extracted from Dex- and vehicle-treated TM cell monolayers using TRIzol reagent (Invitrogen, Carlsbad, CA, USA). RNA preparations were treated with DNase using a DNA-free DNA Removal Kit (Invitrogen) to remove contaminated genomic DNA. First-strand cDNA synthesis was carried out using the SuperScript III First-Strand Synthesis System (Invitrogen) as per the manufacturer’s protocol. Each cDNA synthesis reaction contained the following: 8 µL of total RNA (45 or 90 ng/ µL), 1.25 µM oligo dT, 25 ng of random hexamers, 0.5 mM dNTP, 2 µL of 10X RT buffer, 2.5 mM MgCl 2, 4 µL of 20 mM DTT, 40 units of RNaseOUT, and 200 units of SuperScript III RT. Quantitative PCR was carried out using Dynamite 2X qPCR master mix (Tris pH 8.3, KCl, MgCl2, Glycerol, Tween 20, DMSO, dNTPs, ROX as a normalizing dye, SYBR Green as the detection dye, and an antibody inhibited Taq polymerase; Dynamite is a proprietary mix developed and distributed by the Molecular Biology Service Unit, Department of Biological Science, University of Alberta, Edmonton, Canada). Each qPCR reaction contained 5 µL of SYBR Green master mix, 0.8 nM of each forward and reverse primer, and 2.5 µL of cDNA template. Gene-specific primer pairs used in Q-PCR reactions are listed in the Table.

**Table 1:** Real-time PCR primer pairs for gene expression profiling.

Gene	GenBank accession number	Primers
Myocilin (MYOC)	D88214.1	5’ AGATGCTACCGTCAACTTTGCTT 3’ 5’ CGGTTCTTGAATGGGATGGT 3’
Hypoxanthine phosphoribosyl transferase 1 (HPRT1)	M31642.1	5’ CAGGCAGTATAATCCAAAGATGG 3’ 5’ GTCAAGGGCATATCCTACAACA 3’

The real-time PCR program consisted of an initial cycle of 95 °C for 120 s, 40 cycles of 95 °C for 15 s and 60 °C for 60 s, and a final dissociation curve step. Gene expression was normalized against the expression of HPRT1 (hypoxanthine phosphoribosyltransferase 1), utilizing the 2–ΔΔCT method for data analysis. All qRT-PCR experiments were done in triplicates.

## 3. Results

### 3.1. Culture establishment

Two TM cell strains were cultured from two individual donors: TM-1 (58 years old) and TM-2 (66 years old). The success rate of growth was two cell strains from four dissected eyes of two donors. All the explants that were sandwiched between a gelatin-coated plastic bottom and coverslip without prior collagenase treatment yielded viable cultures (formation of a primary cell culture with the correct cell morphology). Therefore, with a limited number of samples, it was shown that expired tissue might be a source of healthy TM cell cultures. On the contrary, trials to establish primary hTM cultures from donor corneoscleral rims (nontransplantable) by simply placing explants on gelatin-coated plastic bottoms failed, indicating that increased number of anchor points for TM cells facilitates culture establishment. Similarly, none of the collagenase-treated explants resulted in viable cell migration.

### 3.2. Cell morphologies, migration, and population doubling time

**Figure 3:**
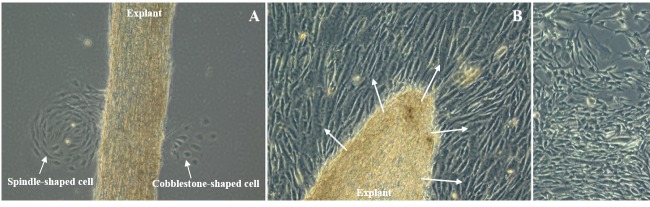
Phase contrast micrographs showing primary TM-2 cell culture. (A) Two-week-old TM explant. Uveal/corneoscleral and JCT cells migrating onto the gelatin-coated polystyrene plate. The confluent primary TM cell populations around the explant (B) and at a distal region from the explant (C) are seen here. Scale bar: 100 μm.

After 14 days, the first cells migrated out of the explants onto the coated substrate (Figure 3A). Cell migration was observed on either side of the explants, suggesting that both beam cells (from uveal and corneoscleral regions) and JCT cells were migrating out of the explants. Furthermore, two cellular morphologies were observed during early stages: cobblestone-shaped cells and spindleshaped cells. However, in subsequent days, the culture substrate was dominated by spindle-shaped cells. Upon reaching confluency, the isolated primary cell culture resembled typical TM cell morphology and formed tightly packed cell monolayers, suggesting that isolated TM cells are contact-inhibited (Figures 3B and 3C).

**Figure 4:**
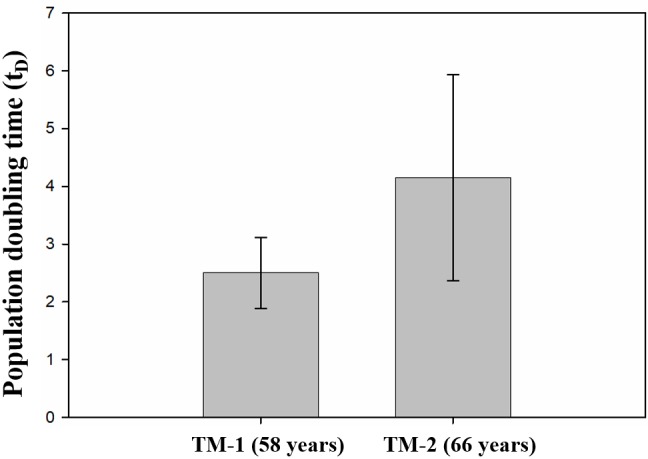
Calculated doubling time of isolated hTM cells (cell passage 1). TM-2 cells showed delayed doubling time compared to the TM-1 (n = 3).

The cell doubling time was calculated under the assumption that the growth rate is proportional to the number of cells in the culture. Since the linear correlation between growth rate and the number of cells would be lost when the culture becomes conuflent, N (number of cells in the culture after 3 days) was determined at semiconfluent stages. The average population doubling time varied between 2.5 and 4 days for isolated hTM cells (Figure 4). When compared to the TM-1 culture (derived from the 58-year-old donor), the growth rate of the TM-2 culture (derived from the 66-year-old donor) appeared to have a delayed doubling time. Additionally, TM-2 cultures exhibited large variations among replicates (Figure 4).

### 3.3. TM cells express ECM proteins

**Figure 5:**
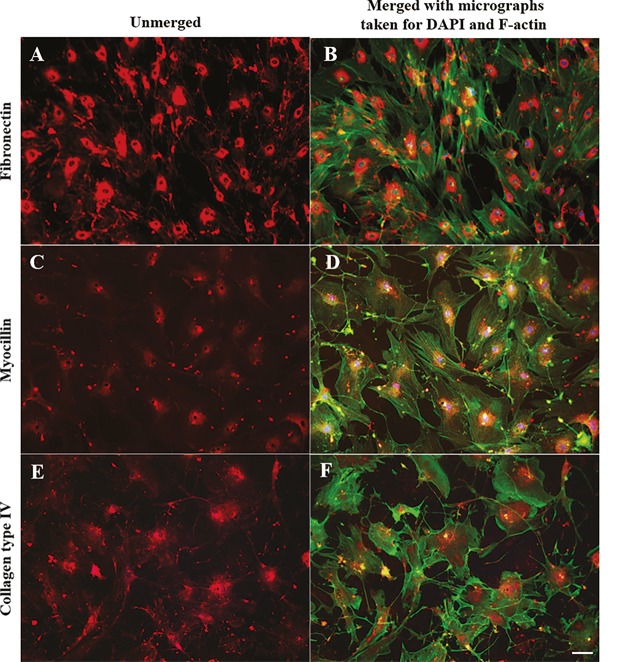
Micrographs of immunostained TM cell monolayers showing F-actins (green), DAPI stained nuclei (blue), fibronectin (A and B; orange), myocilin (C and D; orange), and collagen type IV (E and F; orange). Scale bar: 50 μm.

Immunohistochemical analyses revealed the presence of F-actin filaments in hTM cells exhibiting an organized regular pattern and distributed mainly within cell boundaries. In contrast, fibronectin (FN) was widely distributed throughout the monolayer, exhibiting a more intense immunostaining signal in the perinuclear regions (Figures 5A and 5B). Even without the dexamethasone treatment, isolated hTM cells were immunopositive for myocilin, demonstrating one of the key features of TM cells (Figures 5C and 5D). Myocilin labeling was most intense in the cytosol, particularly in perinuclear regions (Figures 5C and 5D), and intracellular labeling was found to be uniform throughout the preparation. The most intense type-IV collagen (Col IV) labeling was also found in the cytosol (Figures 5E and 5F), resembling the region labeled for myocilin (compared to Figure 5C).

### 3.4. TM cells are phagocytic

**Figure 6:**
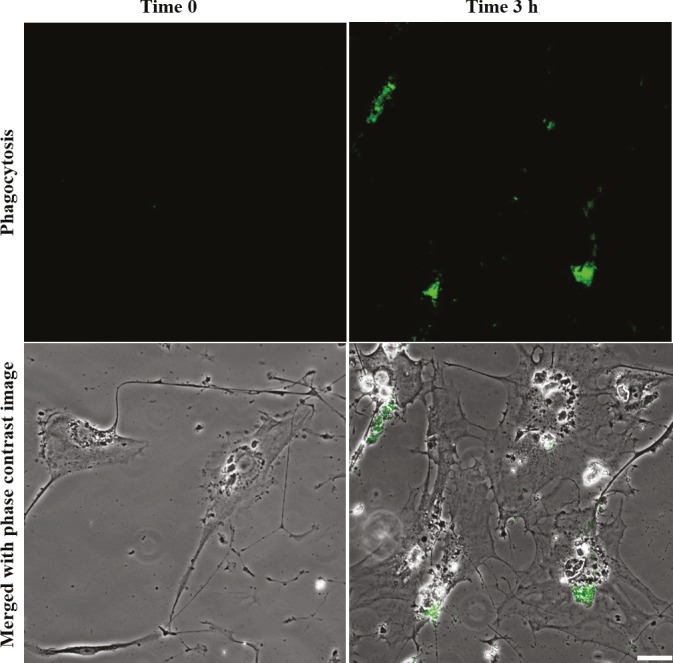
Fluorescence and phase contrast microscopic images showing that isolated TM cells are phagocytic in culture. Fluorescence images (top row) were merged with phase contrast images (bottom row), showing that Staphylococcus aureus bioparticles were actively internalized into TM cells after the incubation period. Scale bar: 50 μm.

hTM P1 (passage 1) cells were plated and challenged with Staphylococcus aureus bioparticles, which emit fluorescence (green) when they are actively internalized. Three hours after being challenged, the hTM cells phagocytosed a high percentage of particles. We found that both TM-1 and TM-2 strains were phagocytic; however, only images taken of TM-1 are shown in Figure 6.

### 3.5. Dex treatment upregulates myocilin expression

**Figure 7:**
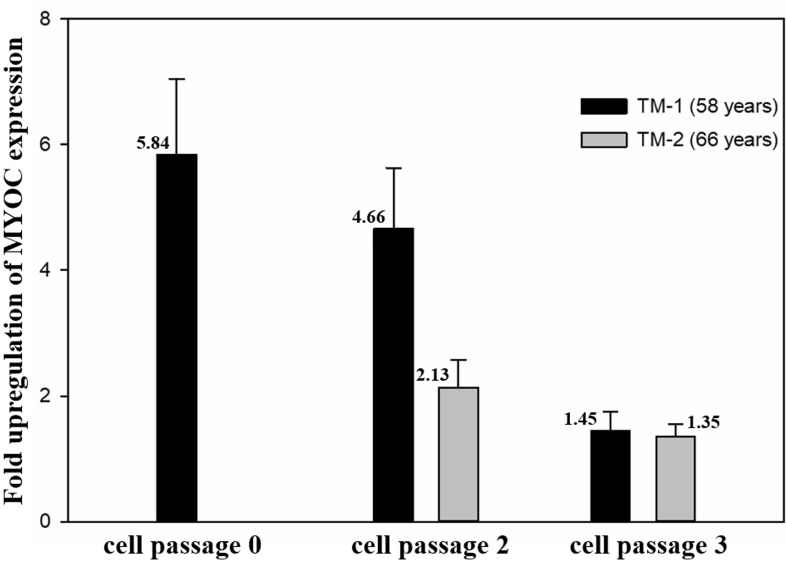
Fold increase in Dex-induced MYOC gene expression in hTM cell cultures (n = 3).

The real-time PCR data indicated that MYOC expression was dramatically upregulated when isolated hTM cells were treated with Dex for 5 days (Figure 7). Notably, the induction of MYOC expression was highly remarkable with a nearly 6-fold difference (compared to ethanoltreated samples) in primary TM-1 cells. However, due to the longer population doubling time and limited cell numbers yielded by the TM-2 cell line, the earliest cell population that we were able to characterize with Dexinduced MYOC expression was passage 2. Despite the fact that these cells were derived from a 66-year-old donor, even the second passage resulted in a nearly 2-fold induction of MYOC expression when cells were treated with Dex. In general, the fold difference in MYOC expression decreased in subsequent passages for both TM-1 and TM-2. At passage 3, Dex-induced MYOC expression in Dex-treated samples was negligible when compared to control samples of both TM-1 and TM-2.

## 4. Discussion

It has been reported that storing ocular tissues in OptisolGS for a prolonged period of time greatly reduces the cell viability as well as cell yield and alters the growth pattern of cells in the culture (Keller et al., 2018) . For instance, Means et al. (1996) demonstrated that epithelial cell viability of cornea stored in Optisol medium is remarkably reduced when stored longer than 6 days. In addition, cell cultures derived from older donors often do not yield an adequate amount of cells as TM cellularity progressively decreases with age (Alvarado et al., 1981) . Therefore, the majority of research on primary hTM cell isolation has been accomplished using donor tissue obtained within 72 h postmortem (Stamer et al., 1995) . However it can be difficult to obtain ocular tissue for research purposes given the high demand for corneal allotransplantation. Therefore, viable and healthy primary cell isolation from nontransplantable tissues would be invaluable. Due to the aforementioned limitations, our attempts to establish hTM cultures from expired ocular tissues using conventional procedures (Polansky et al., 1979; Tripathi and Tripathi, 1982; Stamer et al., 1995) were unsuccessful. Ultimately we were able to isolate, culture, and characterize hTM cells obtained from two donor corneoscleral rims (58 and 66 years old) by placing the explant between cover glass and therefore increasing the anchor points for hTM cells.

The corneoscleral rims used in this study had been stored in Optisol-GS for nearly 16 days before dissection. As our results suggest, sandwiching the explant (not treated by collagenase) between gelatin-coated cover glass and the bottom of the polystyrene plate seems to be a promising approach since only this way were we able to establish primary hTM cultures. The failure of establishing hTM cultures from collagenase-treated explants may be attributed to the loss of cell viability after treatment with collagenase. In hTM cells, the binding of ligands to surface integrin molecules triggers the assembling of focal contacts that in turn initiates various intracellular signaling processes (Zhou et al., 1996) . It has been reported that these adhesive interactions play crucial roles in cell growth, differentiation, and even cell migration (Giancotti, 1997; Howe et al., 1998) . Therefore, it is likely that sandwiching TM explants between two coated substrates encouraged the attachment of more TM cells to cover either the glass or plastic bottom. In addition, placing the TM explants under a coverslip prevented them from floating in the cell growth media and kept them in a permanent place. Therefore, the coverslip allows a more concentrated local environment for the explant, providing a constant and regular supply of oxygen, nutrients, and other growth factors to cells present in the explant, and, in turn, increasing health and growth of TM cells. These factors may have led to the successful establishment of primary cell cultures from our explants. Alternatively, collagen I, collagen IV, fibronectin, and FNC (fibronectin/collagen/albumin) coatings could be considered as potential surrogates for gelatin and can be used to coat culture surfaces to enhance TM cell migration and spreading (Zhou et al., 1996; Engler et al., 2009) .

During the initial stages of primary cell culture establishment, the cells that migrated out from either side of the explant displayed two different morphologies: cobblestone-shaped cells and spindle-shaped cells. As was previously reported, the age of the tissue donor is one of the factors that determine the proportion of endothelial-like cells versus fibroblastic-like (spindle-shaped) cells (Lin et al., 2007; Stamer and Clark, 2017) . At cell confluency, both primary TM-1 and TM-2 cell cultures were consistently dominated by fibroblastic-like cells, as these cultures were derived from older donors. In our study, the population doubling time for TM-1 and TM-2 cell strains at their linear growth phase varied between 2.5 to 4 days, although TM-2 cells appeared to have a small delay in doubling time. When seeding cells (passages 1 and 2) on gelatin-coated polystyrene substrates at a seeding density of 5 × 103/cm2, cultures became conuflent within 1 week. These growth characteristics are comparable with previously reported primary and secondary hTM cell cultures (Alvarado et al., 1981; Stamer et al., 1995; Lin et al., 2007) . Thus, under these culture conditions, cells derived from both the TM-1 and TM-2 cultures could be used for further experiments for at least two passages. In cases where the culture medium is supplemented with aqueous humor or depleted for serum (Fautsch et al., 2005) in order to delay/expedite the proliferation or doubling time, the viable passage number exhibiting the proper phenotype may vary due to the senescence of primary cells.

A unique cell marker has yet to be identified for hTM cells. Therefore we used a variety of markers to characterize the isolated cells including F-actin, fibronectin, collagen IV, and myocilin expression, as well as phagocytic activity and the cell response against glucocorticoid treatment (Gasiorowski and Russell, 2009; Sathiyanathan et al., 2017) . As previously reported, the presence of parallel bundles of actin filaments along the cell’s central axis, known as stress fibers, as well as the presence of cortical actins at the cell periphery are key features of hTM cells (Clark et al., 2005; Murphy et al., 2014) . These fiber networks are believed to be involved in maintaining the contractility of TM cells, which is essential for regulating the aqueous humor dynamics (Murphy et al., 2014; Dufy and O’Reilly, 2018) . In this study, the expression pattern of filamentous actin (F-actin) was found to be similar in both TM-1 and TM-2 cultures. Specifically, actin stress fibers were found to be predominantly aligned along the longitudinal axis of almost all the cells that were studied. In addition, some of the cells also showed circumferentially located actin bundles, resembling cortical actin. Together with alpha smooth muscle actin staining (Supplementary Figure S1), these observations suggest the contractile nature, a characteristic feature of TM cells.

Collagen type IV and fibronectin are two of the most structurally important ECM proteins secreted by hTM cells (Yun et al., 1989; Clark et al., 1994) . As expected, we observed FN distribution in TM cell monolayers on and around the cells. However, we observed that the Col-IV signal was mainly restricted to the cytoplasm. Although these deposits appeared as uneven strands in intercellular spaces as well, their signals were much weaker. As mentioned previously (Dautriche et al., 2014) , collagen type IV is predominantly found in the lining of trabecular beams. Therefore, it is likely that two-dimensional TM cell monolayer cultures do not present favorable conditions for the proper organization of trabecular beams.

TM cells are well known to have phagocytic properties both in vivo and in vitro (Zhang et al., 2007) . The isolated hTM cells in our study clearly and consistently demonstrated a phagocytic capability by internalizing heat-killed Staphylococcus aureus bioparticles into cells, suggesting that cells isolated from older donors can still withstand phagocytic stresses.

Previous studies have shown that MYOC is preferentially expressed in hTM cells (Tamm et al., 1999; Polansky et al., 2000) . Hardy et al. (2005) showed that MYOC protein is mainly localized in intracellular vesicles but can also be found in extracellular spaces associated with secretory pathways. More importantly, MYOC expression is remarkably upregulated when TM cells are treated with glucocorticoids such as dexamethasone, and this feature is not shown by other neighboring cells including SC cells, scleral  fibroblasts, and corneal fibroblasts (Stamer et al., 1998; Polansky et al., 2000) . fibroblasts, dexamethasone-induced MYOC upregulation is the most widely used approach to characterize TM cells. In our study, we observed a similar pattern of MYOC localization as previously reported and an upregulation of the MYOC mRNA level after glucocorticoid treatment. The intense signal observed in the perinuclear region along with the relatively less intense signal observed in the intercellular spaces was consistent with previously reported MYOC localization. Additionally, a dramatic elevation of the MYOC mRNA level was observed when primary TM cells (TM-1 strain) were treated with Dex for 5 days, as compared to the control. However, the level of upregulation induced by Dex was considerably reduced in subsequent cell passages. At passage 3, Dex treatment-induced MYOC expression was negligible in both TM-1 and TM-2 cultures compared to the untreated controls. These data suggest that isolated cells retain their typical TM cell properties until at least cell passage 2.

To the best to our knowledge, this is the first time an expired explant has been utilized in the successful isolation of hTM cells exhibiting various cell-identifying markers. The practical approach applied in this study was to increase the anchor points of the tissue and therefore the cells for proper proliferation. We believe that this method would decrease the demand for fresh donor tissue, which would be appraised for corneal transplant.

## Acknowledgments

The authors would like to thank Jonathan Gotzman for his valuable contributions to the project design and Troy Locke for his technical assistance with the Q-PCR arrays. This work was funded by Alberta Innovates – Technology Futures under Grant Number 20090279/PSI14420-2011. The funder had no role in study design, data collection or analysis, the decision to publish, or the preparation of this manuscript.

## Supplementary Material

Micrograph of immunostained TM cell monolayers showing alpha smooth muscle actins (green) and DAPI-stained nuclei (blue). Scale bar: 100 μm.
