# Supplementary material for: Establishment of human trabecular meshwork cell cultures using nontransplantable corneoscleral rims
Source: Turk J Biol. 2019 Apr 5;43:89–98. doi: 10.3906/biy-1810-69 (PMC6667097; doi:10.3906/biy-1810-69)
Supplement: Micrograph of immunostained TM cell monolayers showing alpha smooth muscle actins (green) and DAPI-stained nuclei (blue). Scale bar: 100 μm. [file turkjbio-43-89-s001.pdf]

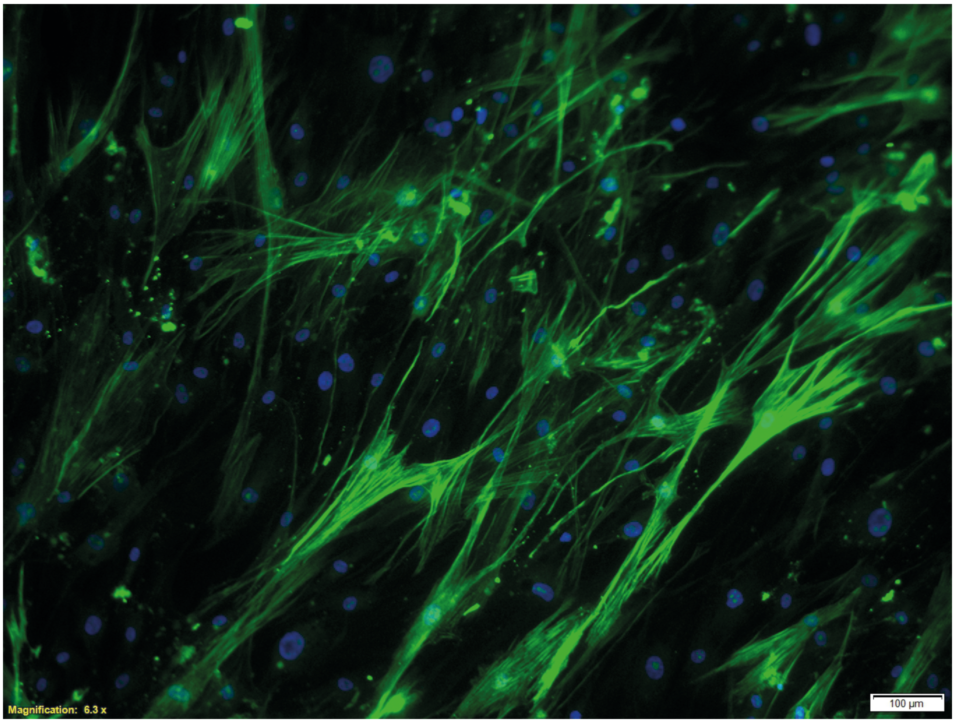


**Supplementary Figure S1.** Micrograph of immunostained TM cell monolayers showing alpha smooth muscle actins (green) and DAPI-stained nuclei (blue). Scale bar: 100 µm.
